# Health-services utilisation amongst older persons during the last year of life: a population-based study

**DOI:** 10.1186/s12877-018-1006-x

**Published:** 2018-12-20

**Authors:** Danielle Ní Chróinín, David E. Goldsbury, Alexander Beveridge, Patricia M. Davidson, Afaf Girgis, Nicholas Ingham, Jane L. Phillips, Anne M. Wilkinson, Jane M. Ingham, Dianne L. O’Connell

**Affiliations:** 1 Department of Geriatric Medicine, Liverpool Hospital, UNSW, Corner of Elizabeth and Goulburn Streets, Liverpool, Sydney, NSW 2170 Australia; 2 Cancer Council NSW, Sydney, Australia; 3 Department of Geriatric Medicine, St. Vincent’s Hospital, and St Vincent’s Clinical School, UNSW, Sydney, Australia; 4 Faculty of Health, University of Technology Sydney, New South Wales, Australia and Johns Hopkins University, Baltimore, MD USA; 5 Centre for Oncology Education and Research Translation, Ingham Institute for Applied Medical Research, South Western Sydney Clinical School, UNSW Australia, Sydney, Australia; 6 Department of Geriatric Medicine, St. Vincent’s Hospital, and UNSW Australia, St Vincent’s Clinical School, Faculty of Medicine, Sydney, Australia; 7 IMPACCT – Improving Palliative, Aged and Chronic Care through Clinical Research and Translation, University of Technology Sydney, Sydney, New South Wales Australia; 8 School of Nursing and Midwifery, Edith Cowan University, Joondalup, WA Australia; 9 UNSW Sydney, Faculty of Medicine, St Vincent’s Clinical School Darlinghurst, Sydney, Australia; 10 Cancer Council NSW, Sydney, Australia

**Keywords:** Geriatric, End-of-life, Death, Hospital-based care, Palliative care, Population-based, End of life care, Linked administrative health data

## Abstract

**Background:**

Accurate population-based data regarding hospital-based care utilisation by older persons during their last year of life are important in health services planning. We investigated patterns of acute hospital-based service use at the end of life, amongst older decedents in New South Wales (NSW), Australia.

**Methods:**

Data from all persons aged ≥70 years who died in the state of NSW Australia in 2007 were included. Several measures of hospital-based service utilisation during the last year of life were assessed from retrospectively linked data comprising data for all registered deaths, cause of death, hospital care during the last year of life (NSW Admitted Patient Data Collection [APDC] and Emergency Department [ED] Data Collection [EDDC]), and the NSW Cancer Registry.

**Results:**

Amongst 34,556 decedents aged ≥70 years, 82% (*n* = 28,366) had ≥1 hospitalisation during the last year of life (median 2), and 21% > 3 hospitalisations. Twenty-five percent (*n* = 5485) of decedents attended ED during the last week of life. Overall, 21% had a hospitalisation > 30 days in the last year of life, and 7% spent > 3 months in hospital; 79% had ≥1 ED attendance, 17% > 3. Nine percent (*n* = 3239) spent time in an intensive care unit. Fifty-three percent (*n* = 18,437) died in an inpatient setting. Hospital records had referenced palliative care for a fifth (7169) of decedents. Adjusting for age group, sex, place of residence, area-level socioeconomic status, and cause of death, having > 3 hospitalisations during the last year of life was more likely for persons dying from cancer (35% versus 16% non-cancer deaths, adjusted odds ratio [aOR] 2.33), ‘younger’ old decedents (29% for age 70–79 and 20% for age 80–89 versus 11% for 90+, aOR 2.42 and 1.77 respectively) and males (25% versus 17% females, aOR 1.38). Patterns observed for other hospital-based service use were similar.

**Conclusions:**

This population-based study reveals high use of hospital care among older persons during their last year of life, although this decreased with increasing older age, providing important data to inform health services planning for this population, and highlighting aspects requiring further study.

## Background

The number of persons aged ≥80 years is projected to more than triple globally by 2050 and the proportion of the population in Australia aged ≥65 years is set to reach 23% by the same date [1, 2]. This population ageing is highly likely to place increasing demands on the existing healthcare structure and support services. The use of healthcare services tends to increase towards the end-of-life, with associated high levels of cost [3–6]. While healthcare utilisation may be more a function of proximity to death, than of age per se, older persons are more likely to die. However, it is possible that during the last year of life older persons may utilise comparatively fewer hospital resources than younger persons. This evidence includes findings suggesting lower rates for hospital admission, intensive care admission, dialysis, and some invasive procedures [3, 5, 7–9]. In an earlier paper by our group of authors, we found that, compared to the reference group aged 60–79, persons older than this (and particularly those aged 90+) had lower levels of healthcare utilisation for some specified measures of healthcare utilisation during the last year of life, including having a lower likelihood of > 3 hospitalisations, prolonged hospitalisation, and time in intensive care [10]. On the other hand, adults aged < 60 years were less likely to experience frequent or prolonged hospitalisation. Given the changing Australian demographic, with increasing numbers of older persons, accurate population-based data regarding hospital-based care utilisation by older persons during the last year of life are needed to better describe the experiences of older people approaching the end-of-life, but to date, such information has largely been lacking [11].

The complicated interplay of many factors influences hospital utilisation towards the end-of-life by older persons. Certain diagnoses, e.g. cancer, have been reported to be associated with increased healthcare use [3, 5–7, 10]. Longer life expectancy may be associated with ‘healthier’ old people, if morbidity is compressed into a shortened time-frame, or frailer older persons who accrue multimorbidty over time [7]. Other factors also may play a role including access to alternative supports, e.g. within the community, enabling older people to be supported outside of the acute hospital setting, and thus impacting on both hospital admission and place of death [12]. For example, in the UK, deaths in hospital for the oldest old declined as the number of nursing home beds increased [6], and in Western Australia, prolonged admissions and death outside of usual place of residence, including for older people, have been attributed, at least in part, to lack of a strongly-developed and accessible community care structure [7, 12]. Service inadequacies, or poorly-met patients’ needs, may underpin prolonged hospitalisations and frequent ED attendances, although increases in primary care involvement may not always translate to reduced hospital utilisation [13]. Importantly, for these and other reasons, patterns of health service ultisation observed for ‘all ages’ cohorts may not apply to older persons [5–7].

The number of persons living to extreme old age (> 100 years) is rising exponentially; it has more than doubled over the past twenty years [14]. Patterns of healthcare needs for this group, who have proven to be robust over multiple decades of life, may be unique. Few studies have specifically explored the end-of-life care and health-services accessed by this cohort [6].

Large population-based studies potentially provide valuable information as those responsible for health services plan for health service development and care for an ageing population. Such studies may also identify gaps where routine health service data collection could be enhanced. In this context, our aim in this current study was to explore patterns of utilisation of acute hospital-based services, during the last year of life for all older residents (aged ≥70 years) of the state of New South Wales (NSW), Australia’s most populous state (estimated population 6,926,990 December 2007) [15], who died in a calendar year using linked, routinely collected administrative health data [10, 16], and to include an analysis of the subgroup aged ≥100.

## Methods

The methods for the overall population study have been described in detail in previous publications [10, 16]. For this current study, analyses of the data set were restricted to decedents aged ≥70 years at the time of death, as follows.

### Data collection, coding and linkage

All deaths registered in NSW in 2007 were identified through the state Register of Births, Deaths and Marriages (RBDM). Coded cause of death was sourced from the Australian Bureau of Statistics (ABS) mortality database, which provided underlying cause of death and contributing causes for January 2007–December 2007 inclusive (after which the ABS ceased to release individual level cause of death records). Cause of death was coded according to the International Classification of Diseases 10th Revision [17]. Decedents were categorised by cause of death according to the underlying cause of death.

Record linkage was performed between datasets, described below, by the Centre for Health Record Linkage (CHeReL) [18] with generation of a linkage key. Health information was detached from personal identifiers to preserve privacy. Data custodians then provided the relevant de-identified data which our research group linked using the project-specific person number provided by the CHeReL Data verification estimated ~ 0.4% false positive and < 0.5% false negative linkages [16].

The linked dataset included data from the NSW Admitted Patient Data Collection (APDC), Emergency Department Data Collection (EDDC) where available (see below) and the NSW Cancer Registry (NSWCR). The APDC included information on admissions to public, private and repatriation hospitals, day procedure centres, including diagnostic, procedural and demographic information. Information on hospital service use covered the period January 2006 to December 2007. Data for the 365 days preceding death were analysed for each person. Cancer diagnoses (1994 onwards), were obtained from the NSW Cancer Registry (NSWCR).

Based on local government area of the person’s place of residence, accessibility to services was defined by the Accessibility/Remoteness Index for Australia (ARIA+) and socioeconomic status quintile was determined using the ABS Index of Relative Disadvantage [19]. While residence in a residential aged care facility (RACF; high or low-level residential care, including nursing homes) was not routinely collected, a surrogate indicator was obtained to identify persons admitted to hospital from and/or discharged from hospital to an RACF. Data regarding whether placement was permanent or temporary were not available.

### Measures of hospital-based healthcare utilisation

Hospitalisations for an individual were analysed by aggregating admissions with overlapping dates, considering them to be part of the one hospital episode.

The EDDC database included 46% (86/185) of Emergency Departments in NSW during the study period covering all major metropolitan EDs, 37 of 39 EDs in the ‘greater Sydney area’, and captured 81% of all ED attendances in NSW in 2007 [16]. Analyses involving ED use were restricted to the greater Sydney area, where the data capture was near complete. The two EDs not included in the EDDC are relatively small facilities, attending to less complex cases. Amongst all 15 NSW local health districts (LHDs), eight were included in the ED data analysis (Central Coast, Illawarra Shoalhaven, Nepean Blue Mountains, Northern Sydney, South Eastern Sydney, South Western Sydney, Sydney and Western Sydney), while seven were excluded due to incomplete data capture (Far West, Hunter New England, Mid North Coast, Murrumbidgee, Northern NSW, Southern NSW and Western NSW).

Measures of interest were selected based on those reported in previous studies [20–24] and through face-face stakeholder meetings and one-one consultations conducted with the project’s investigators and at least 15 clinical health service leaders, analysts and policy makers from the NSW Public Health system. We examined, for each person, the number of hospital episodes, including at least one episode, > 3 episodes; total days in hospital; single prolonged hospitalisations (> 30 days); cumulative hospital in-patient days of > 3 months (> 91 days); number of ED attendances; frequent ED attendance (defined as > 3 visits/year); intensive care unit (ICU) admission; reference to palliative care-related services; and death in an in-patient setting, comprising death in a hospital or stand-alone inpatient hospice/palliative care unit (see below). ‘Prolonged hospitalisation’ included any single hospitalisation > 30 days, with or without intra-hospital or inter-hospital transfers.

Two indicators of palliative-related services were generated [16]. A ‘restricted’ definition included decedents who were documented as having been seen by a specialist palliative care team. This was based on hospital admissions that indicated that the person saw a palliative team, and admissions to any of the five stand-alone inpatient hospice/palliative care units in NSW. A ‘broader’ definition (‘any palliative-related record’) covered all admissions to a hospital facility that were identified as potentially related to palliative care, including those captured by the first indicator and any admission with a diagnosis, patient type or referral code indicating palliative care. In the latter group it was not clear that the palliative care was delivered by a specialist palliative care service - it may have been delivered by another medical team.

Only five stand-alone inpatient hospice/palliative care units, all based in Sydney, have their own unique institution code recorded in the APDC. This is not to say that other hospice or palliative care beds don’t exist. Information regarding palliative care bed usage was limited, as outside of the five stand-alone units, inpatient hospice or palliative care beds were not specifically coded, and at the time there was no comprehensive method of distinguishing between admissions to these beds (which may exist in general hospitals) and admissions to other wards in hospitals.

Arrival/separation status recorded in the APDC and EDDC allowed determination of death in ED or elsewhere (a non-ED location) within the hospital. Deaths occurring in hospital or stand-alone hospice/palliative care facilities were combined for analysis as death in an inpatient setting, and persons who were classified as dead-on-arrival at the hospital or ED were not included as deaths in hospital. APDC data identified ICU admission and duration, but not whether the person was in the ICU at the time of death. For patients with a cancer diagnosis, the NSW Cancer Registry (NSWCR) additionally recorded place of death as inpatient setting, nursing home, or person’s home.

We also examined a number of surgical and percutaneous procedures during the last year of life. These were selected, a priori, as of potential clinical interest in an aged care population, in consultation with a convenience sample of local palliative care and aged care clinician colleagues (*N* = 14), and included, for example, selected major surgical interventions which might carry high risk of morbidity, and some procedures that might be postulated to have limited long-term benefit if a person was at a point in their illness trajectory where symptom relief and palliation had become their sole priority. Hip surgery was defined as total/partial arthroplasty, revision of arthroplasty, or resurfacing. Major bowel surgery included total/partial colectomy, proctectomy, tumour resection (other than endoscopic polypectomy) and pelvic exenteration. Diagnostic tests, e.g. biopsies, endoscopies, and other interventions which are not invasive but which are coded as ‘procedures’ (e.g. imaging and allied health review) were not investigated. Peri-procedural death was defined as death up to 30 days after one of these pre-specified surgical or percutaneous procedures, and prior to discharge.

### Statistical analysis

Statistical analyses were conducted for the whole cohort, and for pre-specified subgroups. For comparison with previously published work in this area, we investigated patient factors, illness-related factors, and environmental factors which might affect healthcare utilisation [6]. In addition to the overall analysis, we additionally performed a pre-specified analysis of the subgroup of decedents aged ≥100.

Associations between selected demographic characteristics and hospital-based healthcare utilisation during the last year of life were investigated using Chi-squared tests, Wilcoxon rank sum tests, and bivariable and multivariable logistic regression, as appropriate. Multivariable models were constructed to include factors which were significantly associated with healthcare use in bivariable analyses, or which have been found to be associated with healthcare utilisation in previous studies, and which were considered biologically or socially plausible contributors to patterns of hospital service use. These included: age group at death (70–79, 80–89, 90+ [reference]), sex (male, female [reference]), country of birth (Australia [reference], other), place of residence (major cities [reference], inner regional, rural [outer regional/remote/very remote]), socioeconomic status quintile of place of residence (most disadvantaged quintile to least disadvantaged quintile [reference]), and cause of death according to the coded underlying cause of death (cancer [reference], other causes). Information about marital status was only available from the APDC and EDDC datasets, so if a decedent did not have linked records in either of these, then this information was not available. We are therefore omitting these variables from analyses where interpretation of the outcome of interest (related to a hospital admission or ED presentation) would require information about persons who were not in these datasets. Decedents with unknown cause of death, i.e. who did not have linked records in the ABS mortality data or NSWCR, were also excluded from the logistic regression analyses (*N* = 1210, 4%), as were the very few with missing values for any of the other factors of interest (*N* = 65, 0.2%). To preserve individuals’ confidentiality, groups (cells) representing ≤5 persons were reported as “≤5” (or as ‘not reported’ where by subtraction of the numbers in the other age categories the remaining group would have a count ≤5).

Statistical analyses were performed using SAS version 9.3 (SAS Institute, NC, USA).

### Ethical approval

The NSW Population and Health Services Research Ethics Committee approved this study (approval number LNR 2012/01/014).

## Results

### Sociodemographic characteristics and cause of death

There were 46,341 deaths in NSW in 2007, among these 13 were excluded as their hospital admission records or ED presentation records could not be reconciled with date of death (for example having multiple admissions recorded after the date of death) and 34,556 decedents were aged ≥70 years at death. Of these 34,556 decedents, 53% were female, 68% were from major cities, and 30% (10,462) were recorded as residing in an RACF (Table 1).

**Table 1:** Demographic characteristics and cause and place of death, decedents aged ≥70 years, NSW, 2007 (*n* = 34,556)

	All aged 70+	Aged 70–79	Aged 80–89	Aged 90+
	*n* (%)	*n* (%)^a^	*n* (%)	*n* (%)
	*n* = 34,556	*n* = 10,252	*n* = 16,354	*n* = 7950
Sex				
Female	18,316 (53)	4165 (41)	8600 (53)	5551 (70)
Male	16,092 (47)	6059 (59)	7696 (47)	2337 (29)
Unknown	148 (0.4)	28 (0.3)	58 (0.4)	62 (0.8)
Place of residence				
Major cities	23,449 (68)	6847 (67)	11,167 (68)	5435 (68)
Inner regional	8308 (24)	2507 (24)	3933 (23)	1868 (23)
Outer regional/Remote/Very remote	2465 (7)	814 (8)	1120 (7)	531 (7)
Unknown	334 (1)	84 (1)	134 (1)	116 (1)
Socioeconomic status				
Most disadvantaged quintile	6492 (19)	2169 (21)	3026 (19)	1297 (16)
Quintile 2	7596 (22)	2539 (25)	3468 (21)	1589 (20)
Quintile 3	7744 (22)	2262 (22)	3695 (23)	1787 (22)
Quintile 4	5939 (17)	1664 (16)	2916 (18)	1359 (17)
Least disadvantaged quintile	6417 (19)	1524 (15)	3099 (19)	1794 (23)
Unknown	368 (1)	94 (1)	150 (1)	124 (2)
Marital status				
Never married	1983 (6)	783 (8)	856 (5)	344 (4)
Married (including de facto)	12,677 (37)	5414 (53)	6040 (37)	1223 (15)
Widowed	13,690 (40)	2300 (22)	6835 (42)	4555 (57)
Separated/Divorced	1523 (4)	805 (8)	605 (4)	113 (1)
Unknown	4683 (14)	950 (9)	2018 (12)	1715 (22)
Country of birth				
Australia	25,671 (74)	7432 (72)	12,194 (75)	6045 (76)
Other country	8657 (25)	2764 (27)	4074 (25)	1819 (23)
Unknown	228 (1)	56 (1)	86 (1)	86 (1)
In residential aged care facility prior to death	10,462 (30)	1952 (19)	5308 (32)	3202 (40)
Cause of death				
Disease of the circulatory system	13,333 (39)	2964 (29)	6547 (40)	3822 (48)
Cancer	8615 (25)	3932 (38)	3769 (23)	914 (11)
Disease of the respiratory system	3127 (9)	936 (9)	1513 (9)	678 (9)
Dementia	1746 (5)	227 (2)	842 (5)	677 (9)
Other known cause	6525 (19)	1868 (18)	3136 (19)	1521 (19)
Unknown cause	1210 (4)	325 (3)	547 (3)	338 (4)
Place of death				
Inpatient setting	18,437(53)	6581 (64)	8770 (54)	3086 (39)
Dead on arrival at ED	835 (2)	401 (4)	361 (2)	73 (1)
Other/sNot recorded	15,284 (44)	3270 (32)	7223 (44)	4791 (60)
For cancer deaths:	*n* = 8615	*n* = 3932	*n* = 3769	*n* = 914
Inpatient setting^b^	5894 (68)	2904 (74)	2493 (66)	497 (54)
Home	1145 (13)	579 (15)	449 (12)	117 (13)
RACF	1240 (14)	350 (9)	660 (18)	230 (25)

*ED* emergency department, *RACF* Residential Aged Care Facility

^a^All *p*-values from a chi-squared test for differences across age groups were < 0.0001, except *p* = 0.002 for “place of residence”

^b^Includes death in hospital, hospice/palliative care ward of a hospital, or stand-alone hospice/palliative care unit

Coded cause of death was available for 96% of decedents aged ≥70 years (95% from ABS, additional 1% from NSWCR). Cause of death was not accessible for 1210 decedents aged ≥70 years, of whom 1160 died in December 2007 as after this time there was a restriction placed on the release of these data. The distribution of known causes of death for the other people who died in December was similar to that for other months. The most common causes of death were circulatory disorders (39% of all deaths) and cancer (25%) (Table 1). Circulatory disease deaths were more common with increasing old age, and cancer deaths were less common (Table 1).

### Hospital and ICU admissions

Overall, 82% (28,366/34,556) of decedents aged ≥70 years had at least one hospitalisation during the year preceding death. Admissions were not limited to the terminal episode, with 66% having been hospitalised even when those episodes concluding with death were excluded (Table 2). The median number of hospital episodes was 2 (interquartile range [IQR] 1–3). One fifth (21%) of decedents aged ≥70 years had at least 1 prolonged hospitalisation (> 30 days). Seven percent of decedents spent more than 3 months in hospital during the last year of life (Table 2). Hospitalisation > 3 months was more common for the ‘younger’ old, and for those resident outside of major cities (Tables 2 and 3). Nine percent (*n* = 3239) of all decedents spent time in an ICU (Table 2).

**Table 2:** Hospital utilisation and presentations to emergency departments, decedents aged ≥70 years by age group (*n* = 34,566)

	All aged 70+	Aged 70–79	Aged 80–89	Aged 90+
	*n* (%)	*n* (%)	*n* (%)	*n* (%)
	*n* = 34,556	*n* = 10,252	*n* = 16,354	*n* = 7950
Hospital utilisation				
≥ 1 hospital episode (%)	28,366 (82)	8977 (88)	13,686 (84)	5703 (72)
- Excluding terminal episode (%)	22,968 (66)	7398 (72)	11,091 (68)	4479 (56)
Median no. of episodes (IQR)	2 (1–3)	2 (1–4)	2 (1–3)	1 (0–2)
> 3 hospital episodes	7223 (21%)	2995 (29%)	3350 (20%)	878 (11%)
Median days in hospital per person (IQR)	17 (3–41)	22 (5–47)	18 (4–42)	10 (0–31)
> 3 months in hospital (%)	2296 (7)	826 (8)	1095 (7)	375 (5)
Had hospitalisation > 30 days (%)	7120 (21)	2236 (22)	3517 (22)	1367 (17)
Spent time in ICU (%)	3239 (9)	1591 (16)	1413 (9)	235 (3)
- Excluding terminal episode (%)	1443 (4)	695 (7)	627 (4)	119 (1)
Restricted Palliative definition^a^ (%)	4488 (13)	2031 (20)	1936 (12)	521 (7)
Broader Palliative definition^b^ (%)	7169 (21)	3102 (30)	3160 (19)	907 (11)
For cancer deaths:	*n* = 8615	*n* = 3932	*n* = 3769	*n* = 914
At least one hospital episode (%)	8165 (95)	3792 (96)	3571 (95)	802 (88)
Median no. of episodes (IQR)	3 (1–4)	3 (2–5)	2 (1–4)	2 (1–3)
Median days in hospital (IQR)	29 (13–51)	31 (15–53)	29 (13–51)	22 (7–44)
Spent time in ICU (%)	796 (9)	470 (12)	289 (8)	37 (4)
Restricted Palliative definition^a^ (%)	3093 (36)	1610 (41)	1263 (34)	220 (24)
Broader Palliative definition^b^ (%)	4673 (54)	2397 (61)	1936 (51)	340 (37)
For non-cancer deaths:	*n* = 25,941	*n* = 6320	*n* = 12,585	*n* = 7036
At least one hospital episode (%)	20,201 (78)	5185 (82)	10,115 (80)	4901 (70)
Median no. of episodes (IQR)	1 (1–3)	2 (1–3)	2 (1–3)	1 (0–2)
Median days in hospital (IQR)	13 (1–36)	14 (2–42)	14 (2–38)	9 (0–29)
Spent time in ICU (%)	2443 (9)	1121 (18)	1124 (9)	198 (3)
Restricted Palliative definition^a^ (%)	1395 (5)	421 (7)	673 (5)	301 (4)
Broader Palliative definition^b^ (%)	2496 (10)	705 (11)	1224 (10)	567 (8)
Death recorded in inpatient setting (%)	18,437 (53)	6581 (64)	8770 (54)	3086 (39)
During terminal hospitalisation^c^				
Restricted Palliative care definition (%)	3455 (19)	1603 (24)	1488 (17)	364 (12)
Broader Palliative care definition (%)	5485 (30)	2433 (37)	2399 (27)	653 (21)
Spent time in ICU (%)	1951 (11)	985 (15)	844 (10)	122 (4)
ED presentations^d^	*n* = 21,544	*n* = 6291	n = 10,269	*n* = 4984
At least one ED presentation (%)	17,117 (79)	5262 (84)	8241 (80)	3614 (73)
Median no. of presentations (IQR)	1 (1–3)	2 (1–3)	2 (1–3)	1 (0–2)
Ever referred to ED from an RACF (%)	3663 (17)	664 (11)	1854 (18)	1145 (23)
Dead on arrival at ED (%)	534 (2)	238 (4)	243 (2)	53 (1)
> 3 ED presentations (%)	3615 (17)	1278 (20)	1751 (17)	586 (12)
Amongst persons resident in RACF prior to death (*n* = 6872)	1674 (24)	402 (31)	888 (26)	384 (18)

*IQR* interquartile range, *ICU* intensive care unit, *ED* emergency department, *RACF* residential aged care facility

^a^Includes persons recorded as undergoing review by a specialist palliative care team or who were admitted to one of five stand-alone hospice/inpatient palliative care facilities in NSW

^b^Patients already captured in restricted definition, plus those referred to palliative care specialist teams or facilities, availing of a palliative care bed, and/or where service category/service-related group/diagnosis code indicated palliative care

^c^Percent of terminal episodes; does not include hospital deaths identified from the NSW Cancer Registry only

^d^For people resident in the geographical area where recording of ED presentations was complete

Note: All *p*-values from a chi-squared test for differences across age groups were < 0.0001

**Table 3:** Factors potentially associated with hospital-based service utilisation, decedents aged ≥70 years, NSW, 2007 (*n* = 34,556)

	%	Unadjusted OR (95% CI)	Adjusted OR (95% CI)^a^	Adjusted *p*-value^a^
≥1 Hospital episode	82			
Male vs Female	87 vs 78	1.89 (1.78–2.00)	1.58 (1.48–1.67)	< 0.0001
Age (OR per 5-year increase in age)^b^		0.74 (0.72–0.75)	0.82 (0.81–0.84)*	< 0.0001
70–79	88	2.77 (2.57–2.99)	1.83 (1.69–1.99)	
80–89	84	2.02 (1.90–2.15)	1.67 (1.56–1.79)	
90+	72	1.00 (reference)	1.00 (reference)	< 0.0001
Socioeconomic quintile (OR per increase in quintile)		0.95 (0.93–0.97)	0.95 (0.92–0.97)*	< 0.0001
Most disadvantaged quintile	85	1.28 (1.17–1.40)	1.30 (1.17–1.44)	
Quintile 2	83	1.09 (1.00–1.19)	1.07 (0.97–1.18)	
Quintile 3	82	1.03 (0.94–1.12)	1.02 (0.93–1.12)	
Quintile 4	82	1.00 (0.92–1.10)	0.96 (0.87–1.05)	
Least disadvantaged quintile	82	1.00 (reference)	1.00 (reference)	< 0.0001
Place of residence: Other vs Major cities	82 vs 83	0.95 (0.90–1.01)	0.87 (0.82–0.94)	0.0002
Cancer vs Non-cancer death	95 vs 78	5.16 (4.67–5.69)	4.29 (3.88–4.74)	< 0.0001
> 3 hospital episodes	21			
Male vs Female	25 vs 17	1.66 (1.58–1.75)	1.38 (1.31–1.46)	< 0.0001
Age (OR per 5-year increase in age^b^		0.73 (0.72–0.75)	0.79 (0.78–0.81)*	< 0.0001
70–79	29	3.32 (3.06–3.61)	2.42 (2.22–2.64)	
80–89	20	2.07 (1.92–2.25)	1.77 (1.63–1.92)	
90+	11	1.00 (reference)	1.00 (reference)	< 0.0001
Socioeconomic quintile (OR per increase in quintile)		1.00 (0.98–1.02)	1.01 (0.99–1.04)*	0.24
Most disadvantaged quintile	22	1.01 (0.93–1.10)	0.96 (0.88–1.06)	
Quintile 2	21	0.97 (0.89–1.05)	0.90 (0.82–0.98)	
Quintile 3	19	0.86 (0.79–0.93)	0.82 (0.75–0.89)	
Quintile 4	21	0.96 (0.88–1.05)	0.91 (0.84–1.00)	
Least disadvantaged quintile	22	1.00 (reference)	1.00 (reference)	< 0.0001
Place of residence: Other vs Major cities	20 vs 21	0.95 (0.90–1.00)	0.95 (0.89–1.02)	0.16
Cancer vs Non-cancer death	35 vs 16	2.78 (2.63–2.94)	2.33 (2.20–2.47)	< 0.0001
Prolonged single hospitalisation (> 30 days)	21			
Male vs Female	22 vs 20	1.16 (1.10–1.22)	1.09 (1.03–1.15)	0.002
Age (OR per 5-year increase in age)^b^		0.93 (0.91–0.95)	0.96 (0.94–0.98)*	< 0.0001
70–79	22	1.34 (1.25–1.45)	1.18 (1.09–1.28)	
80–89	22	1.32 (1.23–1.41)	1.24 (1.16–1.33)	
90+	17	1.00 (reference)	1.00 (reference)	< 0.0001
Socioeconomic quintile (OR per increase in quintile)		1.02 (1.00–1.04)	1.04 (1.02–1.06)*	0.0003
Most disadvantaged quintile	20	0.91 (0.84–0.99)	0.85 (0.78–0.94)	
Quintile 2	20	0.93 (0.86–1.01)	0.86 (0.79–0.94)	
Quintile 3	21	1.00 (0.92–1.08)	0.96 (0.88–1.04)	
Quintile 4	20	0.93 (0.85–1.02)	0.91 (0.84–1.00)	
Least disadvantaged quintile	22	1.00 (reference)	1.00 (reference)	0.002
Place of residence: Other vs Major cities	21 vs 20	1.06 (1.00–1.12)	1.11 (1.04–1.18)	0.002
Cancer vs Non-cancer death	26 vs 19	1.51 (1.43–1.60)	1.45 (1.37–1.54)	< 0.0001
> 3 months total hospitalisation	7			
Male vs Female	7 vs 6	1.11 (1.02–1.21)	1.02 (0.94–1.11)	0.64
Age (OR per 5-year increase in age)^b^		0.86 (0.84–0.89)	0.87 (0.84–0.89)*	< 0.0001
70–79	8	1.77 (1.56–2.01)	1.72 (1.51–1.96)	
80–89	7	1.45 (1.29–1.64)	1.43 (1.27–1.62)	
90+	5	1.00 (reference)	1.00 (reference)	< 0.0001
Socioeconomic quintile (OR per increase in quintile)		0.93 (0.90–0.95)	0.99 (0.95–1.02)*	0.52
Most disadvantaged quintile	8	1.35 (1.17–1.55)	1.07 (0.92–1.24)	
Quintile 2	7	1.23 (1.07–1.41)	0.98 (0.85–1.14)	
Quintile 3	7	1.18 (1.03–1.35)	1.03 (0.89–1.19)	
Quintile 4	6	1.03 (0.88–1.19)	0.97 (0.84–1.13)	
Least disadvantaged quintile	6	1.00 (reference)	1.00 (reference)	0.67
Place of residence: Other vs Major cities	8 vs 6	1.46 (1.34–1.59)	1.41 (1.28–1.56)	< 0.0001
Cancer vs Non-cancer death	7 vs 6	1.16 (1.05–1.27)	1.05 (0.96–1.16)	0.29
ICU admission	9			
Male vs Female	11 vs 8	1.57 (1.46–1.68)	1.29 (1.19–1.39)	< 0.0001
Age (OR per 5-year increase in age)^b^		0.65 (0.63–0.67)	0.65 (0.63–0.67)*	< 0.0001
70–79	16	6.03 (5.24–6.94)	6.05 (5.24–6.99)	
80–89	9	3.10 (2.70–3.57)	3.06 (2.66–3.53)	
90+	3	1.00 (reference)	1.00 (reference)	< 0.0001
Socioeconomic quintile (OR per increase in quintile)		0.94 (0.91–0.96)	0.94 (0.91–0.96)*	< 0.0001
Most disadvantaged quintile	13	1.29 (1.16–1.44)	1.30 (1.15–1.46)	
Quintile 2	9	0.83 (0.74–0.94)	0.84 (0.74–0.95)	
Quintile 3	8	0.81 (0.72–0.90)	0.80 (0.71–0.90)	
Quintile 4	8	0.77 (0.68–0.88)	0.73 (0.64–0.82)	
Least disadvantaged quintile	10	1.00 (reference)	1.00 (reference)	< 0.0001
Place of residence: Other vs Major cities	8 vs 10	0.81 (0.74–0.87)	0.72 (0.66–0.79)	< 0.0001
Cancer vs Non-cancer death	9 vs 9	0.98 (0.90–1.07)	0.72 (0.66–0.79)	< 0.0001
≥1 ED presentation^c^	79			
Male vs Female	83 vs 76	1.56 (1.46–1.67)	1.42 (1.32–1.53)	< 0.0001
Age (OR per 5-year increase in age^b^		0.83 (0.81–0.85)	0.88 (0.86–0.90)*	< 0.0001
70–79	84	1.94 (1.77–2.12)	1.55 (1.41–1.71)	
80–89	80	1.54 (1.42–1.67)	1.37 (1.27–1.49)	
90+	73	1.00 (reference)	1.00 (reference)	< 0.0001
Socioeconomic quintile (OR per increase in quintile)		0.79 (0.77–0.81)	0.80 (0.78–0.82)*	< 0.0001
Most disadvantaged quintile	87	2.68 (2.38–3.02)	2.53 (2.25–2.85)	
Quintile 2	84	2.02 (1.82–2.24)	1.91 (1.71–2.12)	
Quintile 3	83	1.95 (1.76–2.17)	1.90 (1.71–2.12)	
Quintile 4	80	1.57 (1.44–1.71)	1.53 (1.40–1.67)	
Least disadvantaged quintile	72	1.00 (reference)	1.00 (reference)	< 0.0001
Place of residence: Other vs Major cities	82 vs 79	1.16 (1.00–1.35)	1.02 (0.88–1.20)	0.75
Cancer vs Non-cancer death	83 vs 78	1.33 (1.23–1.44)	1.17 (1.07–1.27)	0.0003
> 3 ED presentations^c^	17			
Male vs Female	20 vs 14	1.46 (1.35–1.56)	1.34 (1.24–1.44)	< 0.0001
Age (OR per 5-year increase in age)^b^		0.85 (0.83–0.87)	0.88 (0.86–0.91)*	< 0.0001
70–79	20	1.91 (1.72–2.13)	1.63 (1.45–1.82)	
80–89	17	1.54 (1.40–1.71)	1.41 (1.27–1.56)	
90+	12	1.00 (reference)	1.00 (reference)	< 0.0001
Socioeconomic quintile (OR per increase in quintile)		0.84 (0.82–0.87)	0.86 (0.84–0.88)*	< 0.0001
Most disadvantaged quintile	22	2.02 (1.80–2.26)	1.90 (1.69–2.13)	
Quintile 2	21	1.90 (1.71–2.13)	1.77 (1.58–1.98)	
Quintile 3	16	1.41 (1.25–1.59)	1.37 (1.22–1.55)	
Quintile 4	17	1.45 (1.31–1.61)	1.40 (1.26–1.56)	
Least disadvantaged quintile	12	1.00 (reference)	1.00 (reference)	< 0.0001
Place of residence: Other vs Major cities	21 vs 17	1.32 (1.14–1.52)	1.15 (0.99–1.33)	0.07
Cancer vs Non-cancer death	18 vs 16	1.17 (1.08–1.27)	1.04 (0.96–1.13)	0.33
Palliative care input: broader definition^d,e^	21			
Male vs Female	24 vs 18	1.39 (1.32–1.47)	1.04 (0.98–1.10)	0.21
Age (OR per 5-year increase in age)^b^		0.72 (0.71–0.73)	0.85 (0.83–0.86)*	< 0.0001
70–79	30	3.37 (3.11–3.65)	1.84 (1.68–2.02)	
80–89	19	1.86 (1.72–2.01)	1.37 (1.25–1.49)	
90+	11	1.00 (reference)	1.00 (reference)	< 0.0001
Socioeconomic quintile (OR per increase in quintile)		1.05 (1.03–1.07)	1.02 (1.00–1.05)*	0.05
Most disadvantaged quintile	22	0.93 (0.85–1.01)	1.05 (0.95–1.16)	
Quintile 2	19	0.77 (0.71–0.84)	0.73 (0.66–0.80)	
Quintile 3	18	0.75 (0.69–0.81)	0.73 (0.66–0.80)	
Quintile 4	24	1.03 (0.95–1.12)	1.01 (0.92–1.11)	
Least disadvantaged quintile	23	1.00 (reference)	1.00 (reference)	< 0.0001
Place of residence: Other vs Major cities	18 vs 22	0.74 (0.70–0.78)	0.73 (0.68–0.79)	< 0.0001
Cancer vs Non-cancer death	54 vs 10	11.13 (10.49–11.81)	10.24 (9.63–10.89)	< 0.0001
Death in inpatient setting	53			
Male vs Female	59 vs 49	1.55 (1.48–1.61)	1.31 (1.25–1.37)	< 0.0001
Age (OR per 5-year increase in age)^b^		0.75 (0.74–0.76)	0.80 (0.79–0.82)*	< 0.0001
70–79	64	2.83 (2.66–3.00)	2.17 (2.04–2.31)	
80–89	54	1.82 (1.73–1.92)	1.60 (1.51–1.69)	
90+	39	1.00 (reference)	1.00 (reference)	< 0.0001
Socioeconomic quintile (OR per increase in quintile)		0.92 (0.90–0.93)	0.93 (0.92–0.95)*	< 0.0001
Most disadvantaged quintile	59	1.41 (1.31–1.51)	1.34 (1.24–1.45)	
Quintile 2	56	1.25 (1.17–1.34)	1.17 (1.09–1.26)	
Quintile 3	52	1.08 (1.01–1.15)	1.03 (0.96–1.11)	
Quintile 4	51	1.06 (0.99–1.13)	1.01 (0.94–1.09)	
Least disadvantaged quintile	50	1.00 (reference)	1.00 (reference)	< 0.0001
Place of residence: Other vs Major cities	55 vs 53	1.08 (1.04–1.13)	0.99 (0.94–1.04)	0.62
Cancer vs Non-cancer death	68 vs 48	2.31 (2.20–2.44)	1.96 (1.86–2.06)	< 0.0001

*OR* odds ratio, *CI* confidence interval, *ICU* intensive care unit, *ED* emergency department

^a^Adjusted for sex, age group, socioeconomic quintile (category), place of residence and cause of death, unless otherwise noted

^b^The differences in healthcare utilisation associated with age were similar when age was analysed by age group or by 5-year age increments

^c^Among the 21,544 people resident in the geographical area where recording of ED presentations was complete

^d^Patients already captured in restricted definition, plus those referred to palliative care specialist teams or facilities, availing of a palliative care bed, and/or where service category/service-related group/diagnosis code indicated palliative care

^e^Results were similar for restricted definition of palliative care. (Restricted definition includes persons recorded as undergoing review by a specialist palliative care team or who were admitted to one of five stand-alone hospice/inpatient palliative care facilities in NSW)

*Adjusted for sex, 5-year increase in age, socioeconomic quintile (ordinal scale 1–5), place of residence and cause of death

Hospitalisation was common for all sociodemographic groups. From bivariable analysis, male sex, younger age group, lower area-level socioeconomic group, and cancer death were associated with higher use of several measures of hospital-based service utilisation, while residence outside of major cities was associated with outcomes such as prolonged admission and ED presentation (Table 3). Cause of death differed between those who were not and those who were hospitalised during the last year of life. For example, among non-hospitalised decedents compared to those who were hospitalised, diseases of the circulatory system (50% versus 36%) and dementia (11% versus 4%) were more common and cancer less common (7% versus 29%) (all *p* < 0.0001). Notably, 95% of those dying from cancer were hospitalised at some time during the last year of life, compared to 78% of those dying from non-cancer causes (*p* < 0.001). Differences in cause of death between males and females or residents of major cities or outside major cities were minimal, and of unclear clinical importance (Table 4).

**Table 4:** Characteristics and hospital-based service utilisation decedents aged ≥70, by sex and place of residence

	Females	Males	Major cities	Outside of major cities	*P*-value^a^ (residence)
	*n* (%)^a^	*n* (%)^a^	*n* (%)	*n* (%)	
	*n* = 18,316	*n* = 16,092	*n* = 23,449	*n* = 10,810	
Cause of death					0.002
Disease of the circulatory system	7580 (41)	5753 (36)	8926 (38)	4373 (40)	
Cancer	3786 (21)	4829 (30)	5960 (25)	2645 (24)	
Disease of the respiratory system	1549 (8)	1578 (10)	2168 (9)	952 (9)	
Dementia	1176 (6)	570 (4)	1223 (5)	523 (5)	
Other known cause	3651 (20)	2874 (18)	4483 (19)	2029 (19)	
Unknown cause	574 (3)	488 (3)	689 (3)	288 (3)	
Place of death					< 0.0001
Death recorded in inpatient setting^b^	8889 (49)	9548 (59)	12,433 (53)	5949 (55)	
Dead on arrival at ED	316 (2)	519 (3)	549 (2)	284 (3)	
Other/Not recorded	9111 (50)	6025 (37)	10,467 (45)	4577 (42)	
For cancer deaths:	*n* = 3786	*n* = 4829	*n* = 5960	*n* = 2645	< 0.0001
Inpatient setting^b^	2467 (65)	3427 (71)	4051 (68)	1835 (70)	
Home	503 (13)	642 (13)	756 (13)	389 (15)	
Nursing home	637 (17)	603 (12)	919 (15)	321 (12)	
Unknown place of death	179 (5)	157 (3)	234 (4)	100 (4)	
	*n* = 18,316	*n* = 16,092	*n* = 23,449	*n* = 10,810	
Resident in RACF prior to death^c^	6080 (33)	4382 (27)	7475 (32)	2971 (27)	< 0.0001
Hospital usage					
At least one hospital episode (%)	14,338 (78)	14,028 (87)	19,421 (83)	8875 (82)	0.10
- Excluding terminal episode (%)	11,419 (62)	11,549 (72)	15,895 (68)	7032 (65)	< 0.0001
Median no. of episodes (IQR)	1 (1–3)	2 (1–4)	2 (1–3)	2 (1–3)	0.001^d^
Median days in hospital per person (IQR)	14 (2–38)	21 (5–45)	17 (3–41)	16 (3–42)	0.31
> 3 months in hospital (%)	1167 (6)	1129 (7)	1386 (6)	906 (8)	< 0.0001
Had an episode > 30 days (%)	3586 (20)	3534 (22)	4799 (20)	2311 (21)	0.05
Spent time in ICU (%)	1397 (8)	1842 (11)	2343 (10)	887 (8)	< 0.0001
Restricted Palliative care definition^e^ (%)	2086 (11)	2402 (15)	3631 (15)	851 (8)	< 0.0001
Broader Palliative care definition^f^ (%)	3348 (18)	3821 (24)	5257 (22)	1901 (18)	< 0.0001
ED presentations^g^	*n* = 11,618	*n* = 9928	*n* = 20,296	*n* = 1245	
At least one ED presentation (%)	8849 (76)	8268 (83)	16,099 (79)	1017 (82)	0.04
- Excluding terminal presentation (%)	8564 (74)	7887 (79)	15,473 (76)	977 (78)	0.07
Median no. of presentations (IQR)	1 (1–3)	2 (1–3)	1 (1–3)	2 (1–3)	0.001
> 3 ED presentations (%)	1668 (14)	1947 (20)	3357 (17)	258 (21)	0.0001

Total *N* = 34,556

ED emergency department, *RACF* residential aged care facility, *IQR* interquartile range, *ICU* intensive care unit

^a^Listed p-values represent unadjusted *p*-value for association with place of residence. All p-values for differences by sex were < 0.0001, except *p* = 0.02 for “> 3 months in hospital”

^b^Includes death in hospital, hospice/palliative care ward of a hospital, or stand-alone hospice/palliative care unit

^c^Includes patients who were admitted from or discharged to RACF in year preceding death

^d^Although they have the same median and IQR, the statistical test indicates the distribution of number of episodes was higher for “major cities” than “other”

^e^Includes persons recorded as undergoing review by a specialist palliative care team or who were admitted to one of five stand-alone hospice/inpatient palliative care facilities in NSW

^f^Patients already captured in restricted definition, plus those referred to palliative care specialist teams or facilities, availing of a palliative care bed, and/or where service category/service-related group/diagnosis code indicated palliative care

^g^For people resident in the geographical area where recording of ED presentations was complete

Note: Analyses by sex excluded 148 decedents with unknown sex, analyses by place of residence excluded 297 for whom place of residence was unknown

**Fig. 1:**
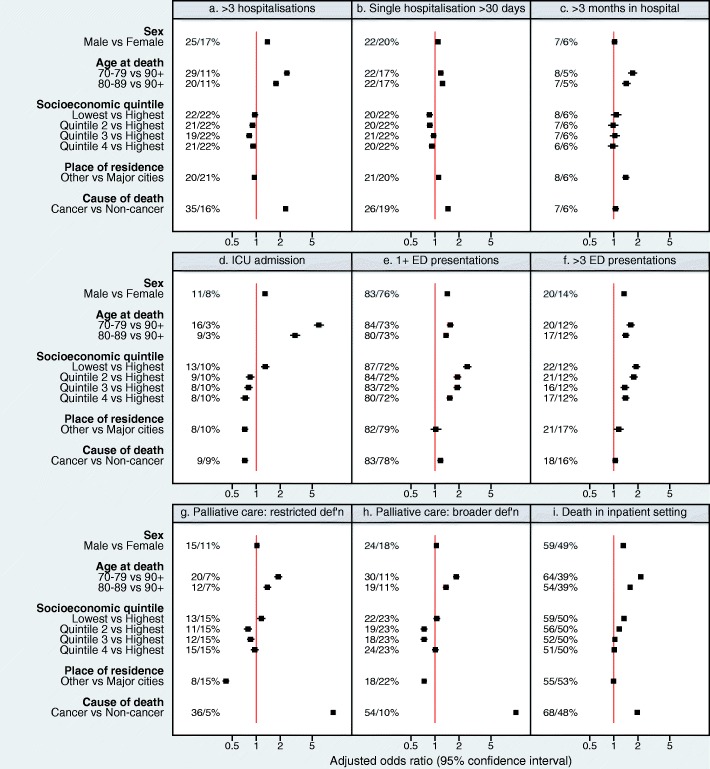
Factors associated with measures of hospital-based service utilisation during the last year of life (*n* = 34,556). Odds ratios and 95% confidence intervals all adjusted for sex, age group, socioeconomic quintile (category), place of residence and cause of death. Panels: (**a**) > 3 hospitalisations, (**b**) single hospitalisation > 30 days, (**c**) > 3 months spent in hospital, (d) ICU admission, (**e**) at least 1 ED presentation, (**f**) > 3 ED presentations, (**g**) Palliative care input (restricted definition), (**h**) Palliative care, broader definition, (**i**) Death in inpatient setting*. ICU: intensive care unit; ED: emergency department. ED measures are for the people resident in the geographical area where recording of ED presentations was complete (*n* = 21,544). Palliative care restricted definition: Includes persons recorded as undergoing review by a specialist palliative care team or who were admitted to one of five stand-alone hospice/inpatient palliative care facilities in NSW. Palliative care broader definition: Patients already captured in restricted definition, plus those referred to palliative care specialist teams or facilities, availing of a palliative care bed, and/or where service category/service-related group/diagnosis code indicated palliative care. * Includes death in hospital, hospice/palliative care ward of a hospital, or stand-alone hospice/palliative care unit. Included as separate file. Figure graphic included in separate file

From multivariable analyses, adjusting for age group, sex, place of residence, area-level socioeconomic status, and cause of death, having > 3 hospitalisations during the last year of life was more likely for persons dying from cancer (35% versus 16% non-cancer deaths, adjusted odds ratio [aOR] 2.33), ‘younger’ old decedents (29% for age 70–79 and 20% for age 80–89 versus 11% for 90+, aOR 2.42 and 1.77 respectively) and males (25% versus 17% females, aOR 1.38). Similar factors were associated with having any hospitalisation during the last year of life (Table 3). ‘Younger’ decedents (22% for 70–79 years versus 17% for 90+, aOR 1.18) and those dying from cancer (26% versus 19% for non-cancer, aOR 1.45) were more likely to experience a prolonged hospitalisation (Table 3). ‘Younger’ decedents were more likely to be hospitalised for > 3 months (8% for age 70–79 versus 5% for 90+, aOR 1.72), as were those resident outside of major cities (8% versus 6% in major cities, aOR 1.41). ‘Younger’ decedents were also more likely to receive intensive care during the last year of life (16% age 70–79 versus 3% for 90+, aOR 6.05), as were males (11% versus 8%, aOR 1.29) and those living in the most disadvantaged areas (13% versus 10% in least disadvantaged areas. aOR 1.30). Those living in regional areas were less likely to be admitted to an ICU than those in the major cities (8% versus 10%, aOR 0.72) (*p* < 0.0001; Fig. 1**,** panels a-d**;** Table 3).

### Emergency department presentations

For decedents from areas included in the ED data collection during the study period, almost four in five (79%, *n* = 17,117) attended an ED at least once during the last year of life (Table 2). The median number of ED presentations during the last year of life was 1 (IQR 1–3). Some had frequent ED attendance with 17% (*n* = 3615) having > 3 ED presentations during the last year of life, and, in this group, the median number of ED presentations was 5 (IQR 4–6). Twenty-five percent (*n* = 5485) of decedents attended ED during the last week of life.

We further attempted to explore how many of ED attendances resulted in admission. In total, 94% were flagged in the EDDC as admitted, but correlating each of these individual episodes with confirmed in-patient episodes on the APDC was beyond the scope of this study.

Twenty-one percent of ED attenders were flagged as being referred to ED from an RACF, while 97% of identified RACF residents had at least one ED attendance during the year prior to death, significantly higher than the non-RACF group (71%, *p* < 0.0001).

The likelihood of presenting to ED during the last year of life was independently associated with living in the most disadvantaged areas (aOR 2.53 vs least disadvantaged), male sex (aOR 1.42) and ‘younger’ old age (aOR 1.55 for age 70–79 and aOR 1.37 for age 80–89, versus aged 90+). Similar associations were noted for those having > 3 ED presentations (aOR 1.90 for most versus least disadvantaged; aOR 1.34 for males vs females; aOR 1.63 for age 70–79 versus age 90+) (*p* < 0.0001 for all) (Fig. 1**,** panels e, f; Table 3).

### Terminal admissions, Palliative Care input, and place of death

Fifty-three percent (18,437/34,556) of decedents aged ≥70 years died in an inpatient setting (Table 1), with 1048 of these dying in one of the five identified stand-alone hospice/palliative care units.

In total, 9% of all decedents aged ≥70 years died in an inpatient setting following prolonged hospitalisation (> 30 days). Of those with terminal admissions > 30 days duration, 58% of patients had no record of hospital-based palliative care (either the restricted or broader definition).

From multivariable analyses, those living outside of major cities were less likely to have mention of hospital-based palliative care (either restricted or broader definitions) (*p* < 0.0001) (Table 3). Mention of palliative care was by far more common for patients who died from cancer (restricted definition: aOR 9.16; broader definition: aOR 10.24, both *p* < 0.0001). Dying in an inpatient setting was more common among decedents who died from cancer (aOR 1.96 vs other causes, *p* < 0.0001), ‘younger’ old decedents (aOR 2.17 for 70–79 years and aOR 1.60 for 80–89 years, versus 90+ years), males (aOR 1.31, *p* < 0.0001) and decedents from the most disadvantaged areas (59% versus 50% least disadvantaged, aOR 1.34, *p* < 0.0001) (Table 3**;** Fig. 1**,** panels g-i).

Further details regarding place of death (in-patient setting, home or RACF) were available only for persons who died from cancer (*n* = 8615). Among this group 13% (*n* = 1145) were recorded as having died at home (Table 1) and those aged 70–79 were most likely to die in an inpatient setting and least likely to die in an RACF, with the opposite true of those aged 90+ (Table 1).

### Selected surgical and percutaneous procedures

We performed a limited analysis of a number of surgical and percutaneous procedures, as detailed above. Fewer than 10 % (8.4%, *n* = 2895) of the total cohort underwent one of these selected procedures during the year prior to death (Table 5), most common among these was coronary angioplasty. Among the 473 decedents who had the one of the procedures classified as “major bowel surgery”, 69% (325) had a diagnosis of bowel cancer. For 187 patients who underwent hip surgery (0.5%), the procedure was elective for 60% (112/187). Two percent (*n* = 706) of all deaths occurred in the peri-procedural period (within 30 days, and prior to discharge) following one of these procedures.

**Table 5:** Selected invasive procedures, decedents aged ≥70 years during last year of life, 2007, NSW (*n* = 34,556)

	All aged 70+	Aged 70–79	Aged 80–89	Aged 90+
	*n* (%)	*n* (%)	*n* (%)	*n* (%)
	*n* = 34,556	*n* = 10,252	*n* = 16,354	*n* = 7950
Percutaneous coronary intervention (PCI)/Stent/Coronary angioplasty	782 (2.3)	420 (4.1)	341 (2.1)	21 (0.3)
Chest drain	597 (1.7)	320 (3.1)	228 (1.4)	49 (0.6)
Major bowel surgery	473 (1.4)	217 (2.1)	226 (1.4)	30 (0.4)
Percutaneous endoscopic gastrostomy (PEG tube)	345 (1.0)	142 (1.4)	168 (1.0)	35 (0.4)
Hip surgery	187 (0.5)	51 (0.5)	111 (0.7)	25 (0.3)
Coronary artery bypass graft (CABG)	161 (0.5)	104 (1.0)	n.r.	n.r.
Nephrostomy	121 (0.4)	57 (0.6)	n.r.	n.r.
Valvuloplasty/Transcatheter aortic valve implantation (TAVI)	94 (0.3)	49 (0.5)	n.r.	n.r.
Valve replacement surgery	91 (0.3)	49 (0.5)	n.r.	n.r.
Spinal decompression/Discectomy	64 (0.2)	44 (0.4)	n.r.	n.r.
Lung resection/Lobectomy	58 (0.2)	41 (0.4)	17 (0.1)	0 (0.0)
Nephrectomy	48 (0.1)	26 (0.3)	n.r.	n.r.
Hysterectomy	45 (0.1)	25 (0.2)	n.r.	n.r.
Craniotomy/Burr hole/Evacuation intracerebral haemorrhage (ICH)	41 (0.1)	27 (0.3)	n.r.	n.r.
Carotid endarterectomy (CEA)	28 (0.1)	15 (0.2)	n.r.	n.r.
Thyroidectomy	27 (0.1)	12 (0.1)	n.r.	n.r.
Mastectomy	20 (0.1)	8 (0.1)	n.r.	n.r.
Prostatectomy	14 (0.0)	8 (0.1)	n.r.	n.r.
Any of the above procedures	2895 (8.4)	1396 (13.6)	1304 (8.0)	195 (2.5)

Presented in order of decreasing frequency

n.r.: not reported, to preserve individuals’ confidentiality

### Persons aged ≥100 years

Of the 360 NSW decedents aged ≥100 in 2007, 80% were female, and one in three were recorded as residing in an RACF. Amongst centenarian decedents, 24% (*n* = 85) died in an inpatient setting, compared with 54% of those aged 70–99 (aOR 0.36, 95% CI 0.28–0.46, *p* < 0.0001)*.* The most common cause of death in this oldest cohort was circulatory diseases (46% versus 39% for 70–99 year olds, *p* = 0.003), while cancer was less frequently the cause of death (5% versus 25% aged 70–99, *p* < 0.0001). “Senility” was recorded as the primary cause of death for 2% (*n* = 6) of this group and was listed among the underlying or contributing causes of death for 11% (39/360). One-third (119) had a record of residing in an RACF during the year prior to death.

Rates of hospitalisation during the last year of life were significantly lower for decedents aged > 100 years: only 51% had any admission (versus 82% for those aged 70–99, *p* < 0.0001), and the median number of days in hospital per person was 1 (IQR 0–18 versus median 17 days for 70–99 year olds; *p* < 0.0001). There were fewer than 5 ICU admissions in this group, which was much lower than the 16% for 70–79 year olds [‘younger’ old]). Only 4% (versus 30%) had mention of any hospital-based palliative care. In the geographical area for which near complete ED data were available, 56% of all decedents aged ≥100 years presented to ED at least once during the last year of life compared with 80% for those aged 70–89; no-one decedent aged ≥100 years was reported as dead on arrival to ED.

## Discussion

Our aim was to describe patterns of utilisation of acute hospital-based services, during the last year of life for all decedents aged ≥70 years who died in NSW in 2007. In this large, population-based, state-wide study, we observed high rates of hospital-based healthcare utilisation by these persons during the last year of life. We found associations between specific decedent characteristics and increased likelihood of service usage, and infrequent mention of palliative care input (although we note below several caveats to this finding). Within this cohort of older decedents, we noted that in the last year of life there were lower rates of hospital utilisation with increasing age, and notably in the “oldest old” (aged ≥100) a group which has been under-investigated to date. We investigated patterns of healthcare utilisation in the last year of life and although the findings are context specific they provide useful information for health services planning and provide a guide for future research for end-of-life care.

Several patient characteristics, such as being male, ‘younger’ old age (age 70–79 more than age 80–89, and age 80–89 more than age 90+), dying from cancer, not living in a major city, and living in the most disadvantaged areas, were associated with an increased likelihood of many outcome measures including any hospitalisation during the last year of life, ≥1 prolonged hospital admission, frequent ED attendance, and time in intensive care. The lower rates of use of hospital-based services in the last year of life with increasing age observed within this cohort of older persons may challenge some perceptions of resource utilisation by older persons. A recent Australian study investigating people with dementia (*n* = 5261) also described higher ED attendance amongst men and ‘younger’ old patients [25]. Other authors have also described higher rates of hospital-based service use by people with cancer [5]. Potentially, females, older persons, and those with higher educational attainment, may be more likely to have prepared or discussed an advance-care plan [26], which might prioritise options other than hospital-based care. However, other factors may also contribute to these findings – including age-dependent access to or rationing of services. Compression of morbidity - where those living to oldest age are fit until very close to death - might also play a part. It is likely that a complex interplay of physical, mental and social vulnerabilities contributes to an increased dependence on emergency services [22, 24] and also to other service use. In many cases, acute hospitalisation is appropriate, but reliance on the acute hospital sector may be exacerbated by a lack of alternatives [7, 24]. While capture of information on RACF status from hospital and ED records may be prone to some bias, 97% of identified RACF residents in our study had at least one ED attendance. In at least a proportion of these cases, acute presentations may be precipitated by lack of or insufficient community-based support structures such as geriatric outreach and community palliative care teams or a lack of relevant expertise within some RACFs [25, 27, 28]. In other studies of patients with dementia, community palliative care has been associated with reduced rates of ED attendance towards the end-of-life [25]. While ED attendance approaching death, and other healthcare interventions, have been identified as potential markers of ‘aggressive’ cancer care [20], these markers cannot necessarily be extrapolated to other older populations with a wide array of diseases other than cancer.

It was uncommon for decedents to have had an admission to an ICU or to have undergone one of the surgical and percutaneous procedures that we explored during the last year of life (Table 5) (both < 10%) in our cohort, and these were even less common with increasing age. However, in performing a limited analysis of a small group of procedures, we acknowledge that this only encapsulates a tiny fraction of all potential procedures which may have been performed. While we note this limitation, further work exploring this area would be important as these and some other procedures, such as mastectomy, coronary artery bypass grafting and craniotomy, might be postulated to be of limited benefit in a setting where end-of-life care and palliation have become the sole priority for the person, as these might also carry significant morbidity. That stated, it is important to note that we cannot interpret these data with regard to the appropriateness or otherwise of such procedures without an understanding of the goals of patient care at the time of the procedure and without any patient-reported outcome measures. Further work in this area is needed.

Only 18% of people were not hospitalised during the year preceding death; this is not dissimilar to findings by other authors [9, 29]. Specific conditions (circulatory or neurological diseases) may be associated with lower likelihood of hospitalisation prior to death, for various reasons [11]. A spontaneous terminal event, without preceding exacerbations, may occur in a previously well person. Lack of hospitalisation may also reflect patients’ preferences, alternative support services, and/or advance-care planning with a focus on community-based care [5, 7, 8, 30]. In our cohort, death from circulatory diseases or dementia was more common amongst decedents who had not been hospitalised during the last year of life. Furthermore, we have assigned cause of death based on the underlying (main) cause of death, and not any of the other contributing factors. Any of the above mentioned factors may contribute to this finding. As the prevalence of dementia rises, and with dementia now the second commonest cause of death in Australia (and commonest for women), healthcare utilisation in this group is likely to become increasingly important. [31] However, the situation is complex. Many more individuals will die with dementia, and not from it, which may also impact on care utilisation moving forward. The fact that hospitalisation in the last year of life was less frequent amongst persons who died from dementia is interesting, because previous data have suggested that individuals with dementia are more likely to be hospitalised than those without [31]. On the other hand, death from circulatory diseases appears to be declining over the past decade, which may represent better primary and secondary prevention, or that other conditions (e.g. dementia and respiratory conditions) are accounting for a greater proportion of coded causes of death [31].

There were challenges detailing palliative care access for this population. We acknowledge that some people may have received palliative care through unrecorded or informal avenues, including care delivered by general practitioners, geriatric medicine teams and palliative care services, before or after admission, and via a diverse range of community palliative care services. The details of the latter were not available or accessible for analysis in this study, and there continues to be no centralised, linkable database for evaluating palliative care service use in NSW. Although the WHO has classified palliative care services at three levels – the ‘palliative care approach’, ‘general palliative care’ and ‘specialist palliative care’ [32] - the data fields and codes available in the data sets used in this study, did not allow us to quantify or distinguish each of these levels. These may range from palliative care delivered by a GP to services delivered by geriatric health services or specialised palliative care teams. We recognise that the availability of palliative care may influence hospital service access but the absence of detailed data limited our ability to interpret the influence of this factor on hospitalisation. However, it remains likely that older people may continue to have unmet palliative care needs towards the end-of-life [5, 33]. Only a fifth of decedents had some form of palliative care input mentioned in their inpatient record, not dissimilar to the 15% of older persons receiving hospice care in an earlier Australian study [7]. We did not limit the concept of palliative care to review by a palliative care team or admission to one of the few stand-alone hospices, and certainly compounding the complexity of comparisons is that definitions of palliative care differ between studies. In Australia, reported rates of palliative care for ‘all ages’ cohorts have varied, ranging from 7% (based on service coding alone) among persons hospitalised in the last year of life [29], to 43%. The latter was reported in one study of hospital- and community-based palliative care service use among decedents with cancer or selected non-malignant conditions that the authors of that study defined as “amenable to palliative care” [34], page 40. In our study, of the 9% who were hospitalised continuously for > 30 days prior to death, mention of palliative care input - which conceivably might have facilitated transition to a non-acute setting - was rare. Inequities in documented palliative care provision were also apparent. Palliative care was less frequently recorded for those residing in regional areas, and those dying from non-cancer causes. NSW Health’s Agency for Clinical Innovation (ACI) has highlighted that even amongst those with non-cancer conditions ‘likely to benefit from palliative end-of-life care’ (e.g. heart failure, dementia), only 4% of decedents accessed inpatient palliative care services [29]. All five stand-alone inpatient hospice/palliative care units in NSW are located in metropolitan areas, with it being acknowledged that those in non-urban areas have been underserved [34, 35]. Evidence indicates that death in hospital is both less attractive to patients, and more costly [5, 12, 36]. While place of death is not the only care consideration for dying patients and their loved ones, proximity to home remains a preference for many [37]. Few patients in this cohort died in a stand-alone hospice/palliative care unit, and while we were unable to clearly identify patients who died in a palliative bed within the acute hospital, based on clinical (anecdotal) experience, it is possible that numbers could have been low. These data highlight the importance of the need for clear and more detailed coding of palliative care service delivery in inpatient records and in health system databases.

The lack of available information about specific input from palliative care specialist services and the absence of community palliative care service data, limited our ability to interpret access to palliative care for this population. However, it is worth noting that at a state level, NSW government, having identified gaps, has recently emphasised the need for an integrated, flexible and responsive approach to palliative care, incorporating primary care and local and networked palliative care specialists within hospital, hospice and community settings [37].

In this population of older decedents, those aged 90+ accounted for 23% of the total cohort, 19% of all bed days and 13% of all hospital episodes. As more people live longer, healthcare utilisation by this group will increasingly impact on healthcare resource requirements, and trends should be monitored over time. Looking at those aged ≥100, we observed lower rates of bed-day usage and hospitalisation episodes in the year before death. This may reflect a different approach to treatment, with fewer hospitalisations, but it is not possible to comment on whether this was appropriate or otherwise. Similar rates of lower hospital-based death have been reported amongst UK centenarians (27%, versus our 24%), with > 50% of those in the UK age group dying in an RACF [6]. In our group, circulatory diseases were the most common cause of death for centenarians, contrasting with “old age” and frailty cited in > 75% of the UK cohort. Whether this represents a real morbidity difference, or cultural practices in death certification is not clear.

Our paper addresses aspects of the end-of-life hospital experiences of people in NSW that have not been reported elsewhere. An earlier paper by our group of authors analysed this data for the whole NSW adult population of decedents, [10] and a recent report by NSW Health’s ACI has also afforded insight into hospital-based service usage in NSW across all ages, but neither of these papers specifically focused on older persons [29]. This paper examines the experiences of decedents from among those in our first study [10] looking specifically and in detail at older persons (≥70 years old) within that population. The ACI report gives detailed information regarding patterns in hospital service use during the last year of life across all age groups for those who had at least one hospital episode [29], but other patient-related factors were not explored in detail, and multivariable analyses were not reported. In this present study, we investigated the impact of other patient-related factors on hospital-based service use in the year before death in persons aged ≥70 years using multivariable analysis. The impact of living in a lower socio-economic area and rural areas, here observed amongst older decedents, as it was previously in decedents of all ages [10], remains of concern in the context of a system of universal health coverage [38].

Strengths of this study include the large population base, comprising all deaths in a calendar year amongst older people in NSW, the most populous Australian state. Comprehensive data collection included several measures of healthcare utilisation, and our findings reflect real-world healthcare use. Linked, de-identified, routinely-collected datasets were analysed to obtain important information regarding current hospital-based healthcare utilisation during the last year of life, and allowed the investigation of multiple factors potentially influencing healthcare utilisation. That stated, despite the rich plethora of data we accessed, our study has some limitations. The ED data collection started with the larger facilities in NSW and has been increasing in coverage over time. As EDDC data were centred on metropolitan areas, we cannot accurately comment on ED attendance in rural areas. Previous authors have also encountered these difficulties, and in some cases have not reported any ED usage data [7]. The NSW ED data collection, has, however, expanded since our study was conducted, with most EDs in the state now contributing to the EDDC, and this may be helpful for future research. We did not have access to cause of death data after December 2007. More recent data have now become available, using an approach that was not established at the time this study was conducted, and could be used for future analyses. Likewise, the incomplete documentation of palliative care services highlights data collection deficits which should be addressed as health systems review the indicators used to capture important information such as palliative care input. Except for those decedents who had a cancer diagnosis, we were unable to access detailed data regarding place of death. Similarly to many epidemiologic studies, we cannot comment on appropriateness of any of this service utilisation. This limitation is compounded by the complete lack of any patient-reported data in the data sets. We are unable to assess whether the needs and priorities of decedents and their families were addressed. Finally, although these data are now some years old (but were the most recent available when we commenced our study), we do not have reason to believe that there have been substantial changes in end-of-life care since the end of the study period. More recent data reported by NSW Health’s ACI indicate that hospitalisation in the last year of life remains common, and that death in the hospital setting, and lack of documented palliative care input, continue to be issues [29].

Analyses of costs, both hospital and non-hospital costs, were beyond the scope of this project. While cost is not the only driver for considering healthcare options at end of life, data from other sources indicate that costs at this time of life are significant [9, 29]. Importantly, we were unable to explore the lived experiences of patients and their carers, individuals’ goals and choices, or quality-of-life aspects of healthcare use during the last year of life. Although we observed high levels of hospital-based healthcare use in this cohort of older persons in the last year of life, we could not explore whether this met patient and carer expectations for end-of-life care. At a government level, policy planning could begin to address this by advocating for the inclusion of patient reported outcome measures - both in care and data sets - and by supporting the funding and adoption of models of care that truly reflect patient/family-centred approaches to care [39].

As detailed above, data capture was incomplete and or/dissatisfactory in a number of areas, including documentation of palliative care provision. There is great potential for health service evaluation to progress significantly if careful attention and consideration could be given at a health system level to the optimal suite of indicators that might routinely be collected to inform evaluation of care at this time of life. As examples, this might include enhanced service-related data - for example more detailed community and inpatient palliative care indicators as well data extracted from patient reported measures as they become more commonly available.

From a research perspective, specific exploration of other aspects of end-of-life care could enhance our understanding of our findings in this study which focussed on specific measures of hospital-based care. These could include, for example, specific exploration of the availability and accessibility of community and social supports (which might influence care settings and use of hospital-based services) and the impact that life-prolonging and symptomatic medications or interventions have on outcomes as these may benefit *or* burden patients towards end-of-life. In addition, focussed studies are needed to explore the needs of potentially vulnerable groups such as people with dementia, those living to extreme old age, and individuals from culturally and linguistically diverse backgrounds. This could be complimented by studies which begin to specifically explore the reasons for some of our findings such as high rates of healthcare service use in certain patient subgroups, evaluation of appropriateness of care, and analyses of patients’ and family caregivers’ experiences and preferences during the end-of-life period.

## Conclusions

This large population-based study reveals high use of hospital care among older persons during their last year of life, although overall this use decreased with advancing old age. The patterns of health care utilisation are striking and our study demonstrates that analyses of linked records utilising established databases can provide powerful indicators of ongoing healthcare utilisation, without the need for significant additional financial investment. The extension and refinement of information recorded in these datasets may prove useful for planning and policy. Rather than a simple increase in existing support services, hybrid or innovative new services may be required. Implementation of longitudinal assessment indicators related to care at this time of life could allow for the impact of healthcare interventions to be monitored over time. In combination, these data will serve to better inform care planning at the population level, and facilitate patient-centred care towards the end-of-life.

## Abbreviations

ABS: Australian Bureau of Statistics
ACI: Agency for Clinical Innovation
aOR: Adjusted odds ratio
APDC: Admitted Patient Data Collection
ARIA: Accessibility/Remoteness Index for Australia
AUD: Australian dollars
CABG: Coronary artery bypass graft
CHeReL: Centre for Health Record Linkage
CI: Confidence interval
ED: Emergency Department
EDDC: Emergency Department Data Collection
ICU: Intensive care unit
IQR: Interquartile range
LHD: local health district
NSW: New South Wales
NSWCR: New South Wales Cancer Registry
OR: Odds ratio
RACF: Residential aged care facility
RBDM: Register of Births, Deaths and Marriages
UK: United Kingdom
